# *In vitro* and *in vivo* reversal of resistance to 5-fluorouracil in colorectal cancer cells with a novel stealth double-liposomal formulation

**DOI:** 10.1038/sj.bjc.6603970

**Published:** 2007-09-11

**Authors:** R Fanciullino, S Giacometti, C Mercier, C Aubert, C Blanquicett, P Piccerelle, J Ciccolini

**Affiliations:** 1 EA3286-Laboratoire de Pharmacocinétique, Université de la Méditerranée, Marseille, France; 2 Department of Medicine, School of Medicine, Emory University, Atlanta, GA, USA; 3 Laboratoire de Pharmacie Galénique, Faculté de Pharmacie, 27 Bd Jean Moulin, Marseille 05 13385, France

**Keywords:** stealth liposome, 5-FU, modulation, resistance, xenografts

## Abstract

Drug resistance is a major cause of treatment failure in cancer chemotherapy, including that with the extensively prescribed antimetabolite, 5-fluorouracil (5-FU). In this study, we tried to reverse 5-FU resistance by using a double-punch strategy: combining 5-FU with a biochemical modulator to improve its tumoural activation and encapsulating both these agents in one same stealth liposome. Experiments carried out in the highly resistant, canonical SW620 human colorectal model showed a up to 80% sensitisation to 5-FU when these cells were treated with our liposomal formulation. Results with this formulation demonstrated 30% higher tumoural drug uptake, better activation with increased active metabolites including critical-5-fluoro-2-deoxyuridine-5-monophosphate, superior inhibition (98%) of tumour thymidylate synthase, and subsequently, higher induction of both early and late apoptosis. Drug monitoring showed that higher and sustained exposure was achieved in rats treated with liposomal formulation. When examined in a xenograft animal model, our dual-agent liposomal formulation caused a 74% reduction in tumour size with a mean doubling in survival time, whereas standard 5-FU failed to exhibit significant antiproliferative activity as well as to increase the lifespan of tumour-bearing mice. Taken collectively, our data suggest that resistance to 5-FU can be overcome through a better control of its intratumoural activation and the use of an encapsulated formulation.

Despite the fact that 5-fluorouracil (5-FU) has been in use for half a century, it remains the gold standard for chemotherapy of colorectal cancer, the third cause of death due to cancer worldwide. With a mere 20% response rate when used as monotherapy, numerous attempts have been made to improve its therapeutic index, both at the bench and at the bedside. 5-Fluorouracil was rationally designed to target thymidylate synthase (TS), an enzyme that is essential for DNA synthesis and cell proliferation; however, the biochemical mechanisms responsible for its antitumour properties are complex and actually require anabolism of this prodrug into specific 5-FU nucleotides within cancer cells. Several enzymes involved in its metabolic activation eventually lead to the formation of active cytotoxic nucleotides or deoxynucleotides (Boyer *et al*, 2004). However, the major mechanism for 5-FU cytotoxicity is the formation of competitive 5-fluoro-2-deoxyuridine-5-monophosphate (FdUMP), thereby inhibiting TS activity with subsequent depletion of intracellular thymidine, suppression of DNA synthesis, and ultimately, apoptosis induction (Pinedo and Peters, 1988; Diasio and Harris, 1989; Langley *et al*, 2003). Overexpression of TS has been demonstrated to be associated with 5-FU resistance in patients with colorectal cancer (Johnston *et al*, 1995; Peters *et al*, 2002). In this respect, controlling the pattern of 5-FU activation, preferentially towards inhibiting TS FdUMP synthesis, is a major goal for optimising its anticancer efficacy.

The key enzyme in the process of yielding intratumoural FdUMP is thymidine phosphorylase (TP), the rate-limiting enzyme in the activation of 5-FU via the DNA pathway (Ciccolini *et al*, 2001; De Bruin *et al*, 2004). Several attempts to boost tumoural TP levels have been published in an effort to improve cell sensitivity to 5-FU or oral 5-FU (capecitabine), by using either ‘Suicide Gene’ strategies (Schwartz *et al*, 1995a, 1995b; Evrard *et al*, 1999; Ciccolini *et al*, 2001), co-treating tumour cells with modulators such as IFN, taxoïd drugs, mitomycine C, or with radiotherapy (Ciccolini *et al*, 2004; Blanquicett *et al*, 2005). Among the numerous compounds tested as putative modulators, 2′-deoxyinosine (d-Ino) is a non-toxic precursor of the TP cofactor, deoxyribose 1-phosphate, that has been shown to enhance 5-FU's antiproliferative activity in several *in vitro* and *in vivo* models, when either used alone or combined with gene therapy strategies targeting TP (Ciccolini *et al*, 2000a, 2000b; Fanciullino *et al*, 2006). So far, extensive erythrocytic metabolism and a failure to improve its pharmacokinetic profile have prevented d-Ino from being considered clinically, as a possible modulator of 5-FU. To achieve this goal, our group previously developed the first encapsulated formulation of d-Ino alone (d-InoL), which was designed to spare it from erythrocytic clearance. This d-InoL proved to increase 5-FU efficacy *in vitro* and in mice xenografts, at doses much lower than the ones used thus far (Fanciullino *et al*, 2005). The purpose of the present study was to develop a novel, stealth double-liposomal formulation encapsulating both 5-FU and its modulator, d-Ino, to enhance further the therapeutic index of 5-FU through a two-pronged strategy: modulation+controlled release.

## MATERIALS AND METHODS

### Cell lines

All experiments were carried out in the 5-FU-resistant, human colon carcinoma cell line SW620 (also known as CCL227), which overexpresses TS. Cells were maintained in RPMI supplemented with 10% fetal calf serum, 5% glutamine, 10% penicillin, 10% streptomycin and 1% kanamycin in a humidified CO_2_ incubator at 37°C. All experiments were performed in exponentially growing cells.

### Drugs and chemicals

Egg yolk phosphatidylcholine (PC), phosphatidylglycerol (PG), cholesterol (C), polyethylene glycol (PEG) covalently binded to phosphatidylethanolamine, 2′-deoxyinosine (d-Ino), 5-FU and 5′-dFUR were all purchased from Sigma (St Quentin, France). Di-kalium hydrogenous phosphate (K_2_HPO_4_) buffer, tetrabutyl ammonium nitrate, acetonitrile, ether and methanol were bought from CarboErba (Milan, Italy). Dimethyl sulphoxide, the apoptosis kit and culture media were purchased from Euromedex (Souffelweyersheim, France). Tritiated 5-FU (12Ci mmol^−1^) was obtained from Moraveck Biochemical (Brea, CA, USA). All reagents were of analytical grade.

### Liposome preparation

Liposomes ((5-FU+d-Ino)-L) were prepared by the classic thin film method (Olson *et al*, 1979). In brief, a lipid mixture composed of egg PC/PG/CHOL/PEG (molar ratios of 7.3 : 0.73 : 1.43 : 0.47) in methanol was evaporated under nitrogen in a round-bottom flask to form a dried thin film. This film was then hydrated with an isotonic carbonate solution (pH 4.2–7.4). The ratio – neutral phospholipid/cholesterol was 30% and when present, the negatively charged lipid was 10% of the neutral lipid. Multilamellar vesicles were formed by vortex mixing the lipid dispersions at room temperature. 5-Fluorouracil (0.2 mM) and d-Ino (0.24 mM) were encapsulated by incubation with the lipid film for 30 min at 37–40°C. The resulting loaded PEG-liposomes were then shaken. Homogenous size distribution as SUV was achieved by 5 min 20 kHz sonication with a probe. To remove the non-encapsulated drug, the liposomal suspension was ultracentrifuged at 70 000 **g** at 4°C for 16 h, and the resulting pellet was re-suspended in either 10 ml of culture media or 10 mM carbonate buffer (pH 7.4), depending on its use (e.g. *in vitro* or *in vivo*). Finally, sterile liposomes were obtained after extrusion through PVDF filters (Durapore 0.22 *μ*m, Millipore, Molsheim, France).

### Polydispersity study

Diameter and particle size distribution were determined by dynamic laser light scattering using a Correlateur RTG submicron particle analyser (Sematech, Nice, France). Measurements were performed at 90° angles, at room temperature. The mean diameter of the liposomes was estimated from the volume distribution curves given by the particle analyser.

### Encapsulation rate and releasing study

Encapsulation rates of both 5-FU and d-Ino were performed by HPLC, using a previously published method (Fanciullino *et al*, 2005). Liposomal release of 5-FU and d-Ino was monitored by dialysis as described elsewhere (Fanciullino *et al*, 2005). Sampling was performed every 30 min, up to 4 h. The total amount of 5-FU and d-Ino released was determined by UV spectrophotometry at 248 nm (Beckman, Villepinte, France).

### Antiproliferative assays

Cells were seeded at a density of 8 × 10^4^ cells per well in 96-well plates. After overnight attachment, exponentially growing cells were exposed to increasing concentrations of 5-FU alone, 5-FU combined with free d-Ino, or the liposomal formulation [5-FU+d-Ino]-L, with gentle shaking for 24 h. Next, drug was removed and the cells were allowed to grow in fresh medium for an additional 48 h. After 72 h of discontinuous exposure, cell viability was evaluated using the classic colorimetric MTT assay (Alley *et al*, 1988). The IC_50_ was defined as the 5-FU concentration inhibiting 50% of cell growth.

### 5-Fluorouracil tumoural metabolism study

Exponentially growing cells were exposed to 1 *μ*M of tritiated 5-FU alone, combined with free d-Ino or as a liposomal preparation [5-FU+d-Ino]-L. After 3, 4 and 6 h exposure, cells were harvested, lysed into 70% methanol and cytosols were isolated by centrifugation (15 000 r.p.m., 30 min) and stored at –80°C until analysis. Separation of 5-FU and its main metabolites was achieved using a HP1090 HPLC system (Agilent, Massy, France) coupled to a Flo-One radioactive detector (Packard, Les Ulis, France) and equipped with an RP18 column (Agilent, France), followed by elution with a K_2_HPO_4_-TBAN/methanol gradient as described previously (Ciccolini *et al*, 2000b).

### Thymidylate synthase inhibition

Thymidylate synthase activity was assessed as described previously (Chazal *et al*, 1997). Briefly, exponentially growing cells were exposed to various combinations of 100 *μ*M of 5-FU alone, 5-FU combined with 250 *μ*M d-Ino, or the liposomal formulation [5-FU+d-Ino]-L for 12 h. Inhibition of TS activity was evaluated at 8, 24, 48 and 72 h. Cells were then harvested and the pellet was stored at −80°C until further analysis. Thymidylate synthase activity was assayed following the standard Roberts' method based on tritiated H_2_O release from [^3^H]deoxyuridine monophosphate, in the presence of excess methylene tetrahydrofolate (Roberts, 1966).

### Cell death induction

Cell-cycle distribution was monitored after exposing the cells for 48 and 72 h to 100 *μ*M 5-FU, 5-FU combined with 250 *μ*M of free d-Ino, or the [5-FU+d-Ino]-L combination at the same concentrations. Cells were washed two times with PBS, trypsinised and suspended in 70% methanol for 1 h, at 4°C. Next, they were centrifuged and immediately collected in 300 *μ*l PBS and 80 *μ*l of propidium iodide (PI), following the manufacturer's recommendations. Samples were analysed with FACScan flow cytometer (Beckman Coulter, Marseille, France) using Cell Quest software. The percentage of cell death was measured by detecting the sub-G_0_/G_1_ peak in PI staining (Derzynkiewicz *et al*, 1992).

### Apoptosis studies

Cells in the exponential phase were exposed to 100 *μ*M 5-FU alone, 5-FU with 250 *μ*M d-Ino, or the liposomal combination [5-FU+d-Ino]-L for 24, 48 and 72 h. Cells were harvested, and early as well as late apoptotic changes were detected by simultaneous staining with Annexin and PI, using an Annexin V FITC staining kit (Sigma, St Quentin Fallavier, France). Cells were treated following the manufacturer's guidelines. FACS analysis was carried out in a FACScan flow cytometer (Becton Dickinson, Poisat, France) using the Cell Quest Software, and apoptosis measured in untreated cells was defined as 100%.

### Drug monitoring study

Comparison of the drug exposure levels achieved in animals treated with the free or encapsulated 5-FU+d-Ino combination was performed. Six-week-old male wistar rats (Charles River, Lyon, France) were kept anaesthetised using O_2_/NO_2_ gas+isoflurane (TEM, Bordeaux, France) during the whole study. Body temperature was maintained at 37°C using a warming blanket. Animals (*n*=3/group) were administered by intraperitoneal injection with 5-FU (50 mg kg^−1^) plus d-Ino (120 mg kg^−1^), either free or combined in the liposomal formulation. Sampling times were as following: T0, T60, T90 and T120 min. One millilitre of blood was withdrawn from jugular vein on heparinised tubes, and plasma was isolated by centrifugation at 5000 **g** for 10 min. Samples were stored at −20°C until analysed. 5-Fluorouracil and d-Ino plasma concentrations were determined by reverse-phase UV–HPLC as described previously, using 2-deoxyadenosine as internal standard (Fanciullino *et al*, 2005). Animal study was performed following animal welfare guidelines, and local animal ethics committee approval was obtained prior to starting the experiments.

### *In vivo* efficacy studies

The antitumour efficacy of 5-FU alone, or in association with free d-Ino, or as the liposomal [5-FU+d-Ino]-L combination was investigated in the SW620 mouse xenograft model. Mouse care was in agreement with the animal welfare guidelines of our institution, and local animal ethics committee approval was obtained prior to starting the experiments. Four-week-old, female Swiss, nude mice (*n*=5 per group, Charles River) were subcutaneously inoculated with 1 × 10^6^ SW620 cells on the right flank. Ten days after implant, and once tumours had reached accurately measurable size, mice were treated with 5-FU by itself, 5-FU combined with free d-Ino, or with the [5-FU+d-Ino]-L form as follows: 5-FU: 50 mg kg^−1^, d-Ino and 120 mg kg^−1^. Drugs were administered intraperitoneally on a 3-times per week basis for 3 consecutive weeks (e.g., D1/D2/D3, D8/D9/D10 and D16/D17/D18). Tumour size was measured three times a week in three dimensions using vernier calipers, and tumour weight (mg) was calculated using the following standard formula: mass=*π*/6 × length × width × height (Waterhouse *et al*, 2005). Preliminary experiments with empty liposomes were conducted to confirm the absence of any *in vivo* antiproliferative activity. Animal weight was monitored as a marker of toxicity. Animals were euthanised whenever a 25% loss of initial weight was observed, or when tumours reached 2500 mg.

## RESULTS

### Encapsulation rate and release studies

**Figure 1:**
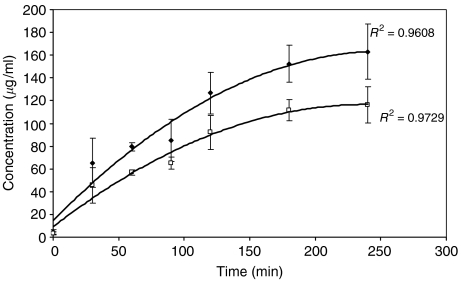
Monitoring of d-Ino (♦) and 5-fluorouracil (5-FU) (□) release from the liposomal [5-FU+d-Ino]-L form. d-Ino and 5-FU concentrations were measured at 248 nm. Bars, s.d.

Homogenous, 100-nm-diameter liposome populations were obtained. Encapsulation rates of 5-FU and d-Ino were 10.6±1.6 and 26.2±5.3%, respectively. Release curves for 5-FU and d-Ino had similar profiles (*n*=5). Both were described by a polynomial equation (Figure 1). No significant difference was observed between the 180 and 240 min concentrations (*P*>0.05, *t*-test). Maximum, 100% release from the liposomes was reached after 4-h incubation for both drugs.

### Modulation of antiproliferative activity

**Figure 2:**
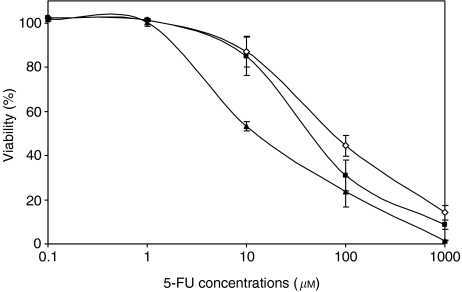
Modulation of 5-fluorouracil (5-FU) (◊) cytotoxicity by free d-Ino (▪) or as the co-encapsulated [5-FU+d-Ino]-L form (▴). Cells were treated for 24 h and viability was measured by MTT testing after 48 extra hours of growth in drug-free medium. Bars, s.d.

Empty liposomes showed no *in vitro* cytotoxicity (data not shown). Results of cytotoxic studies are summarised in Figure 2. Combining 5-FU with either free d-Ino or used as a liposomal [5-FU+d-Ino]-L formulation led to a significant increase in cell sensitivity. The IC_50_s for 5-FU alone, freely associated with d-Ino and encapsulated with d-Ino in a single liposome were 77±6, 57±13 and 48±6.4 *μ*M, respectively (*n*=3). At the IC_50_ level, use of free d-Ino caused a 26% improvement in 5-FU efficacy, whereas the double-agent liposomal formulation caused a 37% increase in SW620 sensitivity. Similarly, cell response was further improved by 52 and 77% (free d-Ino and liposomal formulation, respectively) at IC_20_, and by 18 and 80% (free d-Ino/liposomes) at the IC_80_ levels.

### Modulation of 5-FU intracellular activation

**Figure 3:**
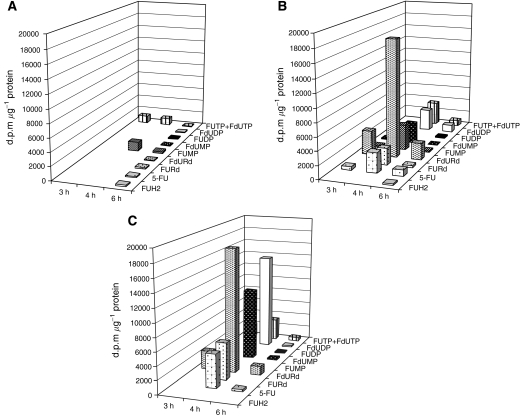
Monitoring of the intratumoural activation of tritiated 5-fluorouracil (5-FU) alone (**A**), associated with free d-Ino (**B**), or as a liposomal [5-FU+d-Ino]-L combination (**C**). Data are from one representative experiment. FUH2=dihydrofluorouracil, FURd=fluorouridine, FUMP=fluorouridine monophosphate, FUDP=fluorouridine diphosphate, FUTP=fluorouridine triphosphate, FdURd=fluorodeoxyuridine, FdUMP=fluorodeoxyuridine monophosphate, FdUDP=fluorodeoxyuridine diphosphate, FdUTP=fluorodeoxyuridine triphosphate.

Intratumoural metabolic profiles of 5-FU used alone, combined with d-Ino or used as a liposomal formulation are displayed in Figure 3. When used alone, 5-FU anabolism took place via the RNA pathway, and little or no FdUMP was formed over the 3–6 h observation period. Combining d-Ino with 5-FU led to a striking change in the activation pattern of 5-FU, with activation occurring predominantly through the DNA pathway, resulting in subsequent intracellular accumulation of fluoro-deoxynucleotides. Overall, anti-TS FdUMP synthesis was increased from 83 d.p.m. mg^−1^ protein (5-FU alone) to 3199 d.p.m. mg^−1^ (5-FU+d-Ino+3801%) and to 11 276 d.p.m. mg^−1^ (liposomal combination, +13 561%). When considering the total cytosolic amount of unchanged 5-FU and metabolites formed, data showed that exposing SW620 cells to the [5-FU+d-Ino]-L combination led to a 36% increase of drug uptake as compared with the free form combination.

### Thymidylate synthase inhibition study

**Figure 4:**
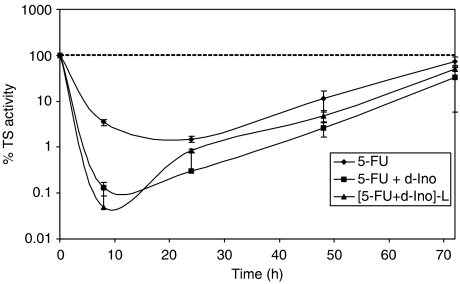
Potentiation of thymidylate synthase (TS) inhibition by 5-fluorouracil (5-FU) alone, combined with free d-Ino or with the liposomal [5-FU+d-Ino]-L association. Cells were exposed for 24 h to 100 *μ*M 5-FU alone, in combination with 250 *μ*M d-Ino or to the encapsulated association.

A significant improvement in TS inhibition was observed both with free d-Ino and with the encapsulated formulation (Figure 4). Thymidylate synthase activity was diminished by 96% after 8 h in cells exposed to 5-FU+d-Ino as compared to 5-FU alone. The liposomal formulation further improved this inhibition level by 61%, with an eventual 98% decrease in TS activity (*n*=3).

### Cell-cycle analysis

**Figure 5:**
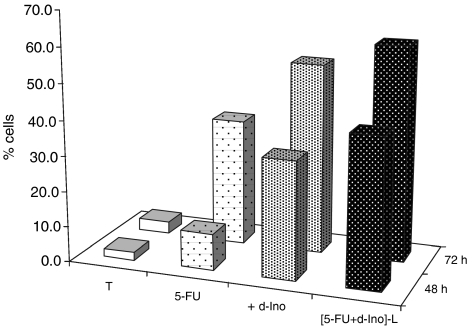
Cell-death induction by 5-fluorouracil (5-FU) alone, combined with free d-Ino or with the liposomal [5-FU+d-Ino]-L association. Cells were exposed for 24 h to 100 *μ*M 5-FU alone or associated with 250 *μ*M d-Ino. Cell death was measured at 48 and 72 h after propidium iodide (PI) staining and flow-cytometry analysis.

Monitoring of the sub-G_0_/G_1_ population at 48 h after PI staining is displayed in Figure 5. Results revealed a 324% higher induction of cell death by 5-FU when associated with free d-Ino, and a 408% increase with the liposomal form, as compared with 5-FU alone. At 72 h, increases in cell death of 150 and 169%, respectively with d-Ino or liposomal formulations (*n*=3) were observed.

### Apoptosis studies

**Figure 6:**
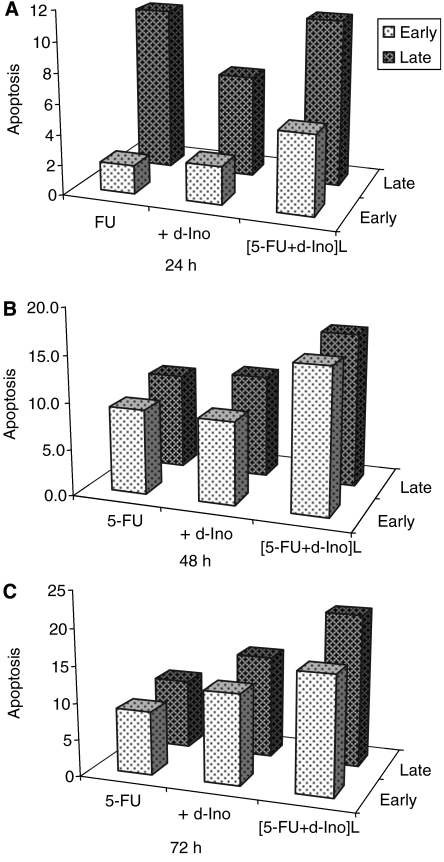
Enhancement of 5-fluorouracil (5-FU)-induced apoptosis. SW620 cells were exposed to 100 *μ*M of 5-FU alone or combined with 250 *μ*M d-Ino, either free or as a liposomal combination. Early and late apoptosis were discriminated by propidium iodide (PI)/Annexin V double staining with subsequent flow-cytometry analysis. Cells were treated for 24 h (**A**), 48 h (**B**) and 72 h (**C**).

A greater induction of both early and late apoptosis was observed in SW620 exposed to FU modulated with free d-Ino, or the co-encapsulated form (Figure 6). Early apoptosis induction was increased by 235, 103 and 136% at 24, 48 and 72 h, respectively (free d-Ino) and by 326, 268 and 219% with the liposomal form, as compared with 5-FU alone. Similarly, late apoptosis was increased by 92, 119 and 138% after 24, 48 and 72 h (free d-Ino) and by 159, 206 and 219% with the encapsulated form, as compared with the use of standard 5-FU (*n*=4).

### Drug monitoring study

Monitoring of 5-FU and d-Ino in plasma was performed after administration of these both drugs, injected either free or as a liposomal combination. Due to analytical interferences with merging endogenous peaks, 5-FU concentrations remained below our limit of detection, regardless of the formulation used. Conversely, d-Ino was fully measurable over the 60–120 min period and showed circulating concentrations up to 139% higher when administered as liposomes, as compared with the free form. At 120 min, 470 ng ml^−1^ of d-Ino was still measured in rats of the liposome group, whereas no modulator was detected anymore in animals treated with the free 5-FU+d-Ino association.

### *In vivo* efficacy studies

**Figure 7:**
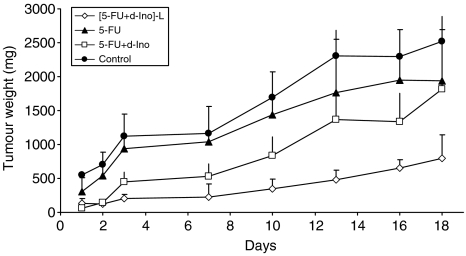
Effects of the liposomal association [5-FU+d-Ino]-L on SW620 tumour growth in nude mice. Animals (*n*=5 per group) were subcutaneously transplanted with SW620 tumoural cells and administered for three consecutive days, over three consecutive weeks with each of the following: carbonate (daily intraperitoneally (i.p.)); 5-fluorouracil (5-FU) (50 mg kg^−1^ daily i.p.) alone or combined with d-Ino (120 mg kg^−1^ daily i.p.), or as the [5-FU+d-Ino]-L formulation (50+120 mg kg^−1^, respectively). Bars, s.d.

**Figure 8:**
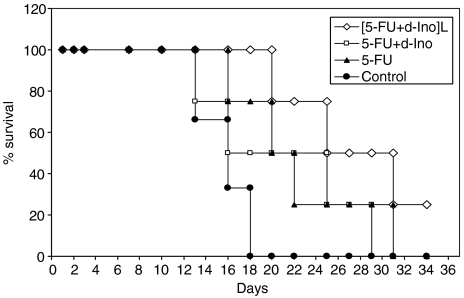
Kaplan–Meier representation of tumour-bearing mice's survival treated either with 5-fluorouracil (5-FU) alone, 5-FU+d-Ino, or with the [5-FU+d-Ino]-L combination, as compared with untreated animals.

Treatment with the empty liposomes showed no impact on tumour growth as compared to untreated animals (data not shown). At the conclusion of the study, tumour size was reduced by 28% (NS), 23% (NS) and 74% (*P*<0.05) in mice treated with 5-FU alone, 5-FU with free d-Ino or the [5-FU+d-Ino]-L formulation, respectively (Figure 7). No signs of toxicity were observed in these animals, regardless of the treatment modality, and no statistical differences were found in animal weights among the different groups (data not shown). When compared to controls, survival time was increased by 25% in the 5-FU-treatment group (20 *vs* 16 days), 56% in the group treated with the 5-FU+d-Ino combination, and by 94% in animals treated with the liposomal formulation (*P*<0.05, Figure 8).

## DISCUSSION

Liposomes can help reduce the toxicity of anticancer drugs, while maintaining or enhancing their efficacy (Di Paolo, 2004). Despite these promising features, with regard to improving of the therapeutic index of anticancer chemotherapies, only doxorubicine (Caelyx®) has been marketed to date as a liposomal formulation. In addition to anthracyclines, several experimental or clinical studies involving encapsulated gemcitabine, methotrexate and platinum derivatives, have been reported over the past 20 years, but thus far, none of them has made its way to the bedside, in a routine, clinical setting (Doddoli *et al*, 2005; Stathopoulos *et al*, 2006; Brusa *et al*, 2007).

Several mechanical and biological mechanisms support the passive tumoural selectivity of liposomes. Sustained release rates, for instance, may enhance antitumour efficacy through an enhanced permeability and retention effect, via a better utilisation of the vascular abnormalities of solid tumours, eventually leading to a greater tumoural uptake (Maeda, 2001). Defects in the capillary endothelium of tumour vasculature are typically in the size range of 200–600 nm and therefore, liposomes of 100 nm in diameter, as generated in this study, can efficiently extravasate and accumulate within the tumour interstitial space, thus providing additional specificity (Yuan *et al*, 1995). Besides their size, intratumoural accumulation of macromolecules is further enhanced by carrier systems displaying reduced release rates and long circulating times (Papahadjopoulos *et al*, 1991; Yuan *et al*, 1994). To slow down the release rate of our liposomes and improve their bioavailability, cholesterol and PEG were used to extend stability via greater membrane rigidity and a ‘stealth’ passage through the liver. Additionally, negatively charged lipids were also used in this study to avoid liposomal aggregation, eventually permitting a better stability. Consequently, drug monitoring of d-Ino revealed circulating concentrations systematically higher when injected as a part of the liposomal formulation, as compared with its free counterpart. Beside these higher plasma levels, sustained exposure to the drug was achieved, as liposomal d-Ino remained fully detectable 2 h after administration, whereas free d-Ino was totally cleared. Despite its extremely widespread use in clinical oncology, few reports have focused on encapsulating 5-FU in simple carriers, probably as a result of its polar and amphotereous properties that render it particularly difficult to entrap in standard liposomes (Simmons and Krame, 1977; Nii and Ishii, 2005). However, as a prodrug, 5-FU presents an opportunity to increase its therapeutic efficacy by combining it with biochemical modulators that can improve its intratumoural activation pattern. Similarly, the strategy of combining encapsulated anticancer drugs with a modulator has been established, for example, in the case of doxorubicin combined with valspodar. However, these two drugs were not encapsulated in a single liposome (Fracasso *et al*, 2005). The specific, well-tolerated modulator, d-Ino, has been identified as a promising agent that can improve the antitumour action of 5-FU (Ciccolini *et al*, 2000b, 2001). Despite significant achievements in enhancing 5-FU efficacy, in various experimental models, *in vivo* handling of d-Ino was rendered difficult as a result of dramatic erythrocytic catabolism. To overcome this, we developed previously the first encapsulated formulation of liposomal d-Ino. We next, demonstrated its ability to modulate 5-FU efficacy in mouse xenografts (Ciccolini *et al*, 2000b). In light of these considerations, the aim of the present study was to reverse 5-FU resistance *in vitro* and *in vivo* through a two-pronged strategy: combining 5-FU with the d-Ino modulator, and treating tumour cells with our new, stealth liposomal formulation comprised of the aforementioned combination. The canonical SW620 line was chosen in this study because it overexpresses TS and, therefore, proves to be highly resistant to 5-FU, thus miming the major cause of treatment failure in clinical settings (Ciccolini *et al*, 2000b). We showed that it was possible to reproducibly co-encapsulate both 5-FU and its modulator, with encapsulation rates and release profiles comparable to the pharmacodynamics of these compounds, showing that intratumoural formation of active FdUMP in the 3–6 h time window was associated with a maximum efficacy in digestive cancer models (Ciccolini *et al*, 2000b, 2001). However, co-encapsulation rates of both 5-FU and its modulator were relatively poor (11 and 26%, respectively). Such a moderate encapsulation rate for 5-FU is not surprising when considering the polar and amphoterous properties of this drug, that render its handling quite difficult when preparing standard pegylated liposomes, as used here (Nii and Ishii, 2005). Indeed, our strategy was to develop a stealth delivery system as basic and as simple as possible, to easily standardise a fabrication process that could be performed in most laboratories equipped with standard apparatus and reagents.

In the current study, encapsulation rates proved to be highly reproducible throughout time (e.g., <2% for 5-FU), thus suggesting little batch-to-batch variation, likely to bias subsequent experiments. *In vitro*, reversal of the resistant profile of our model was achieved, with increases in both cell death and apoptosis induction, as well as marked increases in sensitisation (e.g., +80% at IC_20_) of the SW620 cells to 5-FU. Further experiments confirmed that this increase in efficacy was due to a remarkable switch from the RNA to the DNA activation pathway, with increased formation of active FdUMP when 5-FU was modulated with d-Ino. Subsequent examination of TS activity, as a pharmacological end point, showed profound and sustained inhibition of this target, in cells exposed to the liposomal combination. Additionally, we observed that besides the preferential activation towards FdUMP, a nearly 40% increase in overall 5-FU cytosolic levels was achieved in cells treated with the liposomal formulation, thus probably adding to the higher cytotoxicity effect that was subsequently measured. Interestingly, similar reversal of resistance to 5-FU was also achieved in mice. When used as monotherapy, 5-FU failed to reduce tumour growth as compared with untreated animals. It is noteworthy that combining 5-FU with free d-Ino hardly improved efficacy in this animal study, probably due to the relatively low doses of modulator used (120 mg kg^−1^) and considering d-Ino's dramatic catabolism and the dosage that is normally required (3.2 g kg^−1^) to achieve modulating effects *in vivo* (Ciccolini *et al*, 2000b, 2001). Conversely, [FU+d-Ino]-L caused a 69% reduction in tumour size when compared with untreated animals, and a significant 57% reduction when compared with 5-FU alone. Of note, this increase in efficacy was not accompanied with extra toxicities and all animals showed excellent tolerance, thus indicating an obvious improvement in the therapeutic index of 5-FU. In concordance with the increased antitumoural efficacy and good tolerance, median survival time was nearly doubled in animals treated with our liposomal formulation, compared with standard 5-FU, thus demonstrating that chemoresistance to 5-FU could indeed be overcome.

## CONCLUSION

Drug resistance is a major cause for clinical failure of chemotherapies for digestive cancers. Here, we demonstrate that it is possible to render chemosensitive an experimental model initially highly resistant to 5-FU, a drug used extensively in colorectal cancer. Our dual-agent liposomal formulation caused, through a more effective activation of the 5-FU prodrug into active metabolites interfering with TS, an increase in the induction of both cell death and apoptosis. When examined in tumour-bearing mice, this new formulation led to a striking improvement in treatment efficacy and subsequent survival in animals, thus suggesting that reversal of chemoresistance could be achieved following our combined (encapsulation+modulation) strategy.
